# Lipidomics-Based Investigation of the Effects of Ginsenoside FI on Free Fatty Acid-Induced Metabolism in HepG2 Cells

**DOI:** 10.3390/ph19050772

**Published:** 2026-05-15

**Authors:** Jie Zhou, Dai-Feng Su, Yu-Xin Chi, Quan-Cheng Chen, Yu-Xin Huang, Shu-Xian Chen, Shuang Liu, Jin-Hao Liu, Wei-Yun Zhang

**Affiliations:** 1 Xiamen Key Laboratory of Traditional Chinese Medicine Bioengineering, Traditional Herbal Culture Inheritance Research Center, School of Pharmacy, Xiamen Medical College, Xiamen 361023, China; 2240320433@fjmu.edu.cn (J.Z.); sudaifeng@fjmu.edu.cn (D.-F.S.); 17359852293@163.com (Y.-X.C.); 2250320416@fjmu.edu.cn (Y.-X.H.); chenshuxian@xmmc.edu.cn (S.-X.C.); 2 Fujian Provincial Key Laboratory of Innovative Drug Target Research, School of Pharmaceutical Sciences, Xiamen University, Xiamen 361002, China; chenqc@xmu.edu.cn; 3 Jilin Jinziyuan Biotechnology Co., Ltd., Siping 136400, China; jhssjt@163.com (S.L.); jh124635@163.com (J.-H.L.)

**Keywords:** lipidomics, metabolic disorders, ginsenoside F1, HepG2 cells

## Abstract

**Objective:** This study investigated the effects of ginsenoside F1 on lipid metabolism using cell-based assays combined with lipidomics. **Methods:** The optimal non-cytotoxic concentration of ginsenoside F1 was determined by the CCK-8 assay. A hyperlipidemic cell model was established by inducing HepG2 cells with free fatty acids (FFAs). Model cells were treated with ginsenoside F1 (0.2 µM, 0.8 µM, and 3.2 µM) or simvastatin (3.2 µM, positive control) for 24 h. Intracellular lipid accumulation was determined by measuring absorbance at 510 nm, together with quantification of total cholesterol (TC) and triglyceride (TG) contents. Untargeted lipidomics was employed to explore alterations in the lipid profile and identify relevant metabolic pathways. **Results:** Compared with the model group, lipid deposition, total cholesterol, and triglycerides were significantly reduced by ginsenoside F1 (*p* < 0.05). A total of 110 differential metabolites, mainly phosphatidylinositol and phosphatidylcholines, were identified by lipidomics, with glycerophospholipid and ether lipid metabolism highlighted as the key regulated pathways. Potential roles of targets including Akt1, PPARG, and EGFR, as well as pathways related to cancer and lipid metabolism, were further indicated by network pharmacology and molecular docking. **Conclusions:** FFA-induced lipid disorders in HepG2 cells were alleviated by ginsenoside F1, potentially through the regulation of glycerophospholipid metabolism.

## 1. Introduction

Hyperlipidemia is characterized by elevated levels of one or more lipids in the plasma, such as total cholesterol (TC), triglycerides (TGs), and low-density lipoprotein cholesterol (LDL-C), or decreased high-density lipoprotein cholesterol (HDL-C). It is a disease of dysregulated lipid metabolism in the body and a high-risk factor for atherosclerosis in vital target organs such as the heart and brain [1,2]. Current primary medications for hyperlipidemia include statins, fibrates, niacin, and intestinal cholesterol absorption inhibitors. While these drugs demonstrated definite effects, long-term use is often accompanied by side effects such as gastrointestinal adverse reactions, chronic liver and kidney function damage, and rebound after discontinuation, most of which are unavoidable [3,4,5]. Statin therapy for hyperlipidemia can easily induce abnormal liver function or even hepatotoxicity [6], in addition to adverse reactions such as increased blood glucose level [7] and renal damage [8], significantly affecting patient prognosis. Therefore, effective therapeutic drugs with fewer side effects are urgently needed.

**Figure 1:**
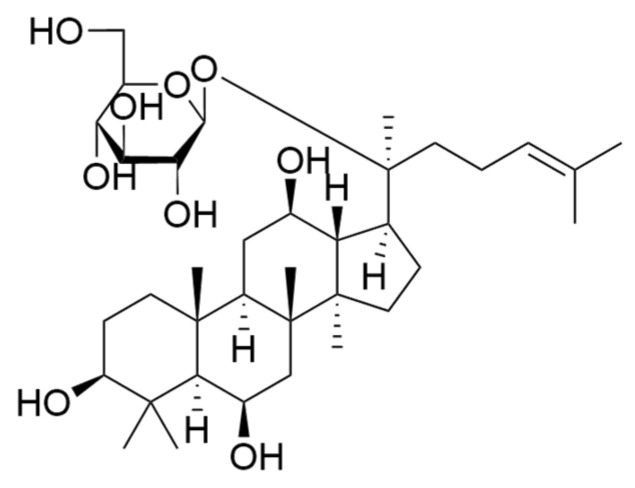
Chemical structure of ginsenoside F1.

In traditional Chinese medicine theory, lipids are regarded as essential substances derived from qi, blood, and body fluids [9,10]. The research on TCM for hyperlipidemia has gradually increased. New possibilities for its treatment are provided by TCM, which is characterized by multi-system and multi-target actions, with which body functions are holistically regulated, lipid metabolism disorders are ameliorated, and fewer adverse reactions are caused [11]. Currently, TCMs used to treat hyperlipidemia mainly include *Ginseng Radix Et Rhizoma*, *Notoginseng Radix Et Rhizoma*, *Radix Astragali*, etc. [12]. *Ginseng Radix Et Rhizoma*, traditionally recognized as a precious medicinal herb and an authentic medicinal material from Northeast China, has long been honored as the “King of Herbs.” With a long history of being used for both medicinal and edible purposes, it has been demonstrated to be effective in treating various diseases in clinical practice. Additionally, it has been widely applied in medicinal diets, health products, and functional foods [13]. For example, ginseng is one of the main ingredients in the famous nourishing prescription “Qiongyu Gao”, which has a long history of use in China and is efficacious in strengthening the body and prolonging life. A range of pharmacological properties have been substantiated through clinical and preclinical studies, such as the regulation of metabolic abnormalities, modulation of immune function, anti-tumor action, and protection of the cardiovascular and cerebrovascular systems [14]. Ginsenoside F1 (Figure 1) is a triterpenoid saponin and a member of the ginsenoside class, the key constituents of Ginseng Radix Et Rhizoma. Since disordered cholesterol metabolism is closely associated with hyperlipidemia and systemic lipid metabolism disturbances, the reported activity of ginsenoside F1 in improving cholesterol metabolism disorders suggests its potential for ameliorating hyperlipidemia. Existing studies have indicated that ginsenoside F1 regulates cholesterol homeostasis through the SREBP2/HMGCR pathway [15] and can also reduce lipid accumulation by activating the cAMP/PKA/HSL signaling pathway to upregulate UCP1 expression [16]. However, its systematic regulatory mechanism and comprehensive effect on FFA-induced hepatic lipid metabolism disorders have not been fully elucidated. The aim of the present study was to explore the lipid-regulating activity of ginsenoside F1 and its correlation with lipid metabolism. Therefore, the hyperlipidemic cell model was established. The degree of fat accumulation in the HepG2 cells induced by oleic and palmitic acid after ginsenoside F1 intervention was measured after the intracellular lipid content, TG content, and TC content were determined by a microplate reader. Simultaneously, lipidomics was utilized for the preliminary screening and analysis of differential lipid metabolites in different groups. Integrated with network pharmacology and molecular docking, these methods were applied to further study the metabolic pathways, as well as the mechanism of ginsenoside F1 in the lipid metabolism of oleic- and palmitic-acid-induced HepG2 cells for accumulation model. Therefore, this study was expected to provide key insights into clarifying its lipid-lowering activity and underlying mechanism of action.

## 2. Results

### 2.1. Effect of Ginsenoside F1 on HepG2 Cell Proliferation/Toxicity

The cell viability was normalized to that of the control group, which was designated as 100%. Compared with the control group, HepG2 cell viability was slightly decreased by 3.2 μM ginsenoside F1, while it was slightly enhanced by 0.2 μM and 0.8 μM ginsenoside F1. However, no statistically significant difference in cell viability was observed in any ginsenoside F1 group compared to that in the control group, with viabilities remaining above 90%. This demonstrated that HepG2 cell growth and proliferation were not significantly inhibited by 0.2 μM, 0.8 μM, and 3.2 μM ginsenoside F1.

### 2.2. Detection of the Effect of Ginsenoside F1 on Lipid Content in HepG2 Cells Induced by Oleic Acid and Palmitic Acid

**Figure 2:**
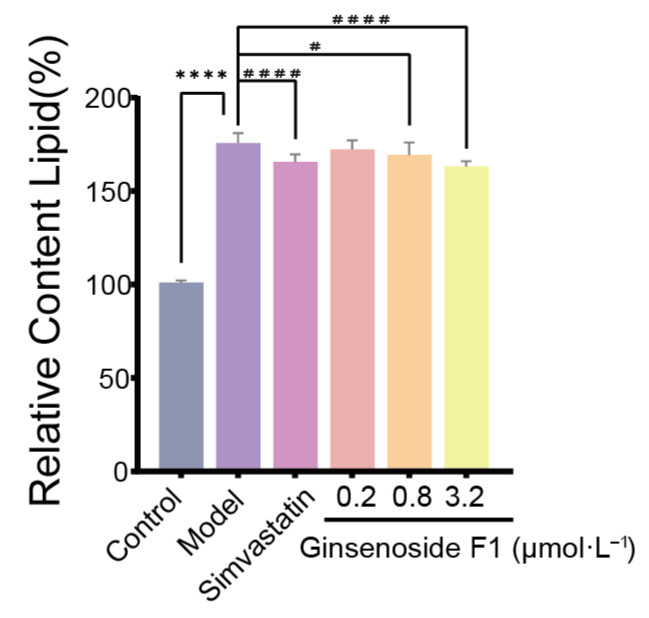
Effect of ginsenoside F1 on relative lipid content in HepG2 cells induced by oleic and palmitic acid (*n* =3). Note: Compared with the control group, **** *p* < 0.0001; ^#^ *p* < 0.05. Compared with the model group, ^####^ *p* < 0.0001. The bars represent different groups: gray (Control group), purple (Model group), light purple (Simvastatin group), pink (0.2 μM Ginsenoside F1 group), light orange (0.8 μM Ginsenoside F1 group), and light yellow (3.2 μM Ginsenoside F1 group).

Quantitative analysis of cellular lipids was conducted after treatment with free fatty acids (FFAs) (Figure 2). With the lipid content of the control group set to 100%, the relative lipid content in the model group was found to be significantly increased compared with that of the control group (*p* < 0.01), indicating that the hyperlipidemic cell model was successfully established. Compared with that of the model group, the relative lipid content was significantly reduced by treatment with 0.8 µM and 3.2 µM ginsenoside F1 (*p* < 0.05). No significant reduction in lipid content was observed in the 0.2 µM ginsenoside F1 group relative to that in the model group.

### 2.3. Effect of Ginsenoside F1 on TG and TC Content in HepG2 Cells Induced by Oleic Acid and Palmitic Acid

**Figure 3:**
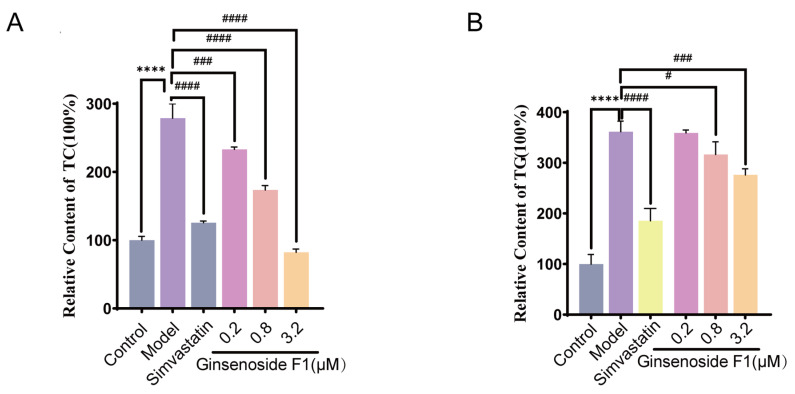
Effect of ginsenoside F1 on relative contents of TG and TC in HepG2 cells induced by oleic and palmitic acid (*n* = 3). (**A**) Relative TC content; (**B**) Relative TG content. The bars represent different groups: gray (Control group), purple (Model group), light gray/yellow (Simvastatin group), light pinkish-purple (0.2 μM Ginsenoside F1 group), light pink (0.8 μM Ginsenoside F1 group), and light orange (3.2 μM Ginsenoside F1 group). Note: Compared with the control group, **** *p* < 0.001. Compared with the model group, ^#^ *p* < 0.05, ^###^ *p* < 0.001, ^####^ *p* < 0.0001.

As shown in Figure 3, compared with those in the control group, the relative contents of TG and TC in the model group were markedly elevated (*p* < 0.001), suggesting that the hyperlipidemic cell model was successfully established. The TC content in the 0.8 µM and 3.2 µM ginsenoside F1 groups was markedly decreased compared with that in the model group (*p* < 0.0001), indicating a possible inhibitory effect of ginsenoside F1 on TC accumulation. The TG contents in these two treatment groups were also significantly different from that in the model group (*p* < 0.05), suggesting that ginsenoside F1 may reduce intracellular TG accumulation. In contrast, no statistically significant effect on TC or TG content was observed in the 0.2 µM ginsenoside F1 group relative to that in the model group.

### 2.4. Multivariate Statistical Analysis of the Lipidome of HepG2 Cells Induced by Oleic Acid and Palmitic Acid After Ginsenoside F1 Intervention

**Figure 4:**
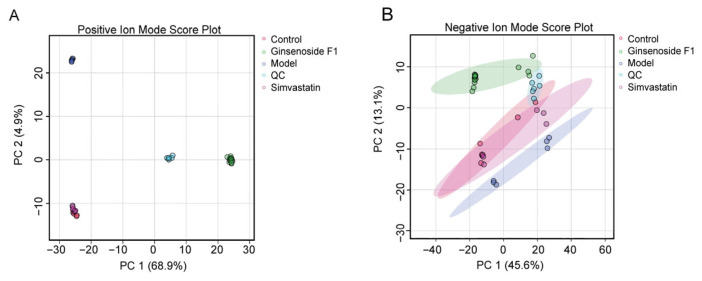
PCA analysis of cell samples. Note: (**A**) represents the positive ion mode. (**B**) represents the negative ion mode.

Principal component analysis (PCA), an unsupervised multivariate statistical method, was employed to extract significant information from the multivariate data matrix and to reveal the metabolic distinctions among groups. As shown in Figure 4, the quality control (QC) samples in both positive and negative ion modes were tightly clustered, indicating satisfactory instrument reproducibility. In the positive ion mode, distinct intra-group aggregation was observed in the control, model, and ginsenoside F1 groups, with pronounced inter-group separation, suggesting substantial changes in lipid metabolites among the different HepG2 cell groups. In the negative ion mode, although samples within each group were well-clustered, clear differentiation between groups was not achieved.

**Figure 5:**
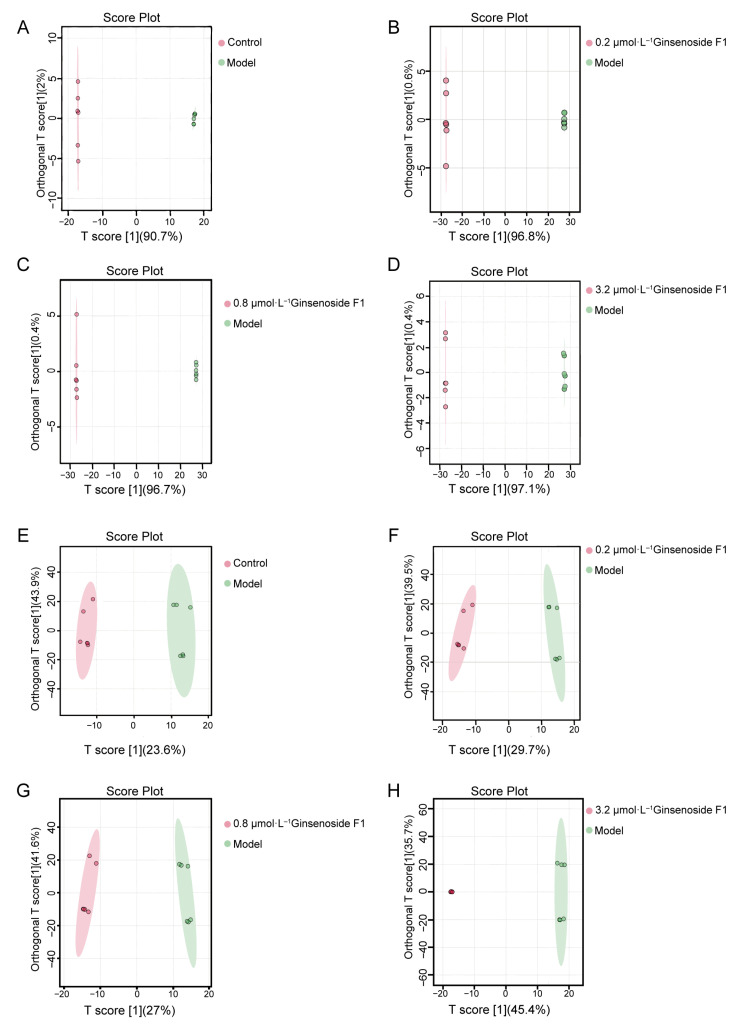
OPLS-DA analysis of cell samples under positive and negative ion modes (*n* = 6). Note: (**A**–**D**) represent the positive ion mode. (**E**–**H**) represent the negative ion mode.

A more accurate prediction between two sample groups can be achieved by OPLS-DA analysis under the supervised mode, with the inter-group variations being effectively reflected. In the OPLS-DA score plots (Figure 5), clear separation from the model group was observed for all experimental groups in both positive and negative ionization modes, with clustering of biological replicates within each group. Distinct lipidomic profiles between each experimental group and the model group were collectively demonstrated by these results.

To address concerns about overfitting and validate the reliability of the OPLS-DA models, permutation tests (*n* = 100 permutations) were conducted, with key model parameters listed in Table 1. In both positive and negative ionization modes, all cross-validated models yielded *Q*^2^ values > 0.4, demonstrating robust predictive performance and clear separation between groups at the current sample size (*n* = 6 per group). While permutation tests confirmed that the observed *R*^2^*Y* and *Q*^2^ values were significantly better than those obtained from random permutations (*p* < 0.01), supporting the presence of true biological differences, we acknowledge that the small sample size (*n* = 6 per group) inherently increases the risk of overfitting in multivariate models. Therefore, these results should be interpreted with caution, and further validation with larger sample sizes would be desirable to confirm our findings.

**Figure 6:**
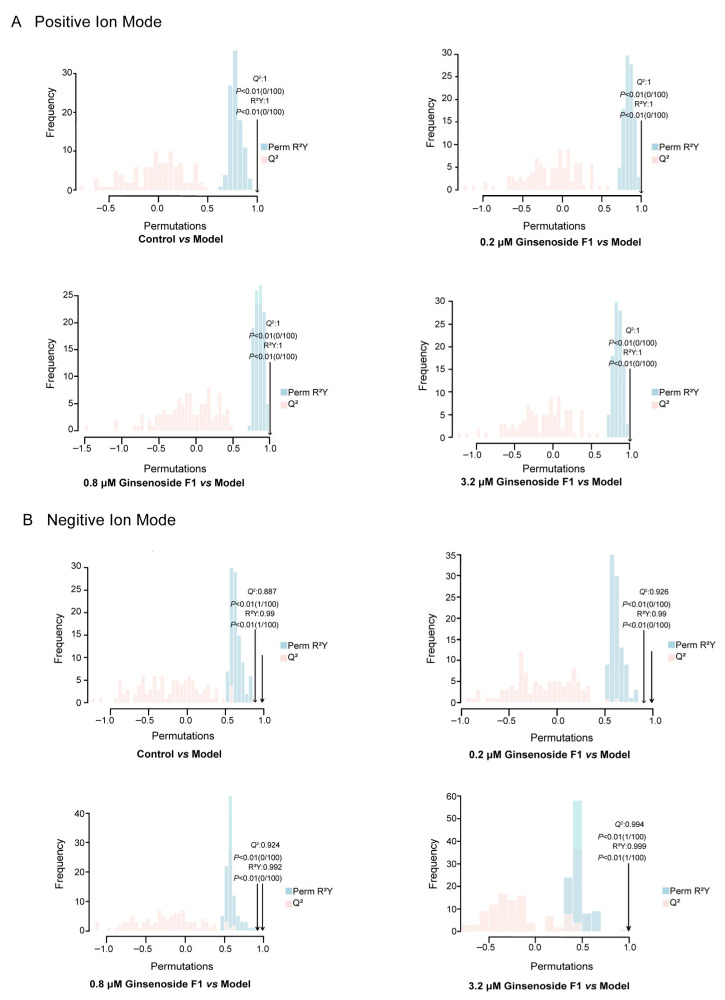
Permutation tests of cell samples under positive and negative ion modes. Note: (**A**) represents the positive ion mode. (**B**) represents the negative ion mode.

The *Q*^2^ intercepts of all models after 100 permutation tests were less than 0.05. The absence of overfitting in any group model was demonstrated, which attests to the reliability of the model data (Figure 6). Given the relatively small sample size (*n* = 6 per group), the cross-validated evaluation parameters should be interpreted with caution. While the positive-ion-mode OPLS-DA models for each group showed high apparent stability and predictive performance, with *R*^2^*Y* values close to 1 and *Q*^2^ values well above the conventional threshold of 0.4, these metrics are prone to overestimation in small datasets. The permutation tests confirmed no formal overfitting risk, yet the high *R*^2^*Y* and *Q*^2^ values likely reflect the small sample size and limited statistical power, rather than perfect separation of biological variation.

### 2.5. Screening and Analysis of Differential Lipid Metabolites in HepG2 Cells Induced by Oleic Acid and Palmitic Acid After Ginsenoside F1 Intervention

**Figure 7:**
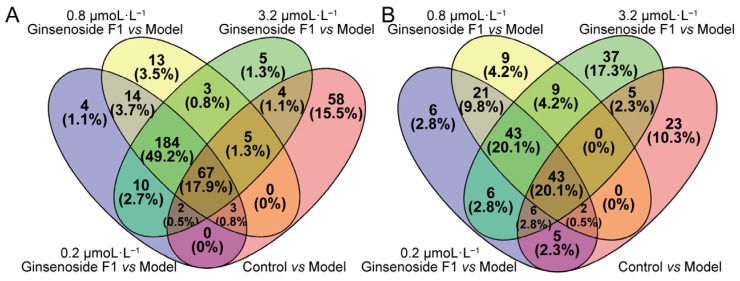
Venn diagram of differential metabolites after ginsenoside F1 intervention. ((**A**) represents the positive ion mode; (**B**) represents the negative ion mode.)

Given the small sample size in each group (*n* = 6), the OPLS-DA models presented relatively high fitting performance, which may lead to overestimated group discrimination. Therefore, differential lipid screening was interpreted with caution and further verified by combining multivariate and univariate statistical analyses. To screen out differential lipid components, lipids were annotated and compared across groups based on the combined criteria of VIP > 1.0, fold change (FC) ≥ 2.0 or < 0.5, and *p* < 0.05. The potential differential lipid metabolites among different groups were visualized by Venn diagrams. As demonstrated in Figure 7A,B, a total of 67 overlapping differential lipid metabolites were identified in positive ion mode and 43 in negative ion mode, with a combined total of 110 unique differential lipids from the two ionization modes.

**Figure 8:**
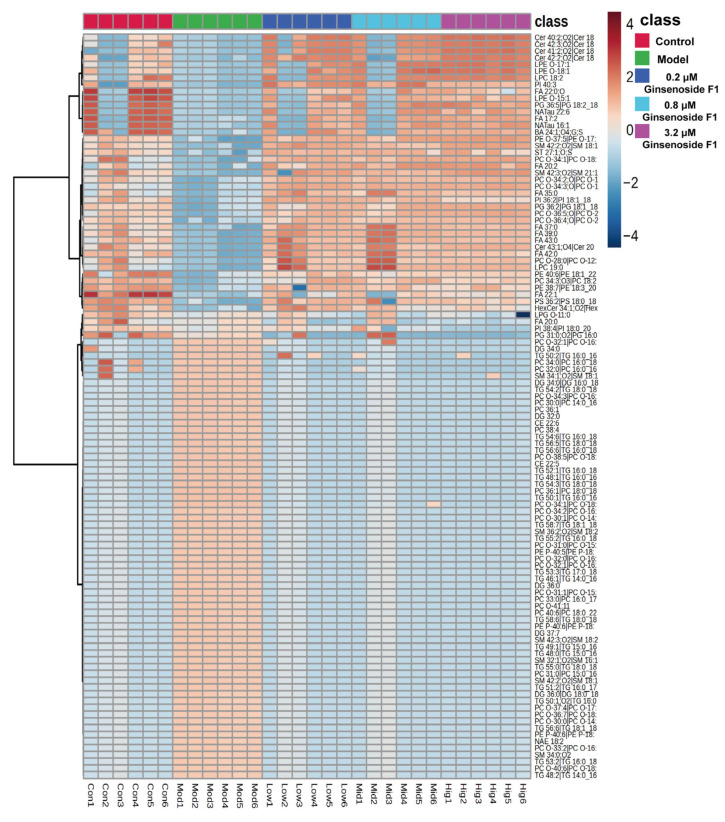
Clustering heatmap of differential lipid metabolites related to HepG2 cells induced by oleic and palmitic acid after ginsenoside F1 intervention.

The common differential lipid metabolites among groups were composed mainly of triglycerides and phosphatidylcholines. To visually compare the changes of these differential metabolites in each group, their relative peak areas were converted into a visual heatmap and subjected to cluster analysis. As exhibited in Figure 8, red represents increased concentration, and blue represents decreased concentration. The major differential lipids between each concentration of the ginsenoside F1-treated group and the model group were identified as 51 glycerophospholipids (GPs), 10 fatty acyls (FAs), 28 glycerolipids (GLs), 13 sphingolipids (SPs), 3 sterol lipids (STs), and saccharolipid. Among these, a relatively high proportion of the differential lipids were accounted for by glycerophospholipids, which were thus established as the main differential lipid category. These 110 differential lipids may represent crucial metabolic markers of ginsenoside F1 in treating lipid metabolism disorders.

### 2.6. Lipid Metabolic Pathway Analysis of HepG2 Cells Induced by Oleic Acid and Palmitic Acid After Ginsenoside F1 Intervention

**Figure 9:**
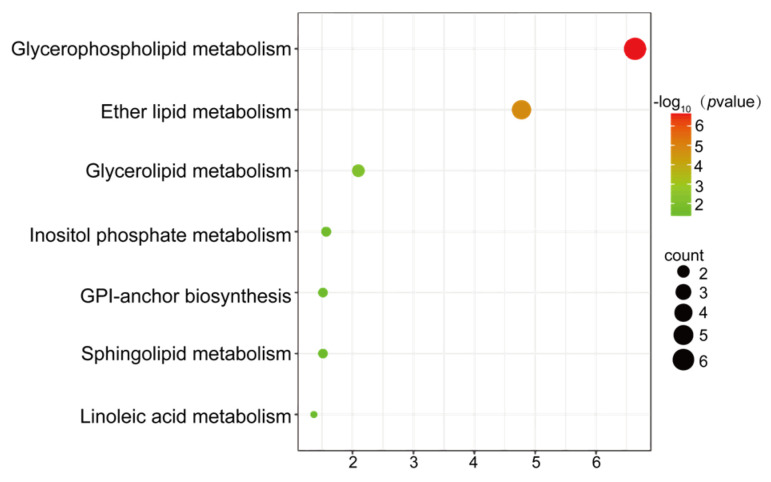
KEGG enrichment analysis of differential lipid metabolites related to HepG2 cells induced by oleic and palmitic acid after ginsenoside F1 intervention. These metabolic pathways, along with their associated detailed information, could be accessed via the KEGG: Kyoto Encyclopedia of Genes and Genomes (https://www.genome.jp/kegg/) accessed on 27 August 2025.

Pathway enrichment analysis of the 110 differential lipid metabolites was conducted using the Metaboanalyst 6.0 database. The importance of a pathway and the significance level of the pathway enrichment analysis were evaluated by the impact value and -log *P* of the metabolic pathways, respectively. Only pathways meeting *p* < 0.05 were retained [17]. These differential lipids were mainly involved in the following metabolic pathways, such as glycerophospholipid metabolism, ether lipid metabolism, glycerolipid metabolism, phosphatidylinositol signaling system, glycosaminoglycan biosynthesis, sphingolipid metabolism, and linoleic acid metabolism. Among the enriched pathways, glycerophospholipid metabolism was highlighted as the most significant pathway, based on the lowest *p* value and the highest pathway impact value in the KEGG enrichment analysis (Figure 9). The expression of differential metabolites in the glycerophospholipid metabolism pathway was summarized in Table 2. It was revealed by the enrichment analysis of these pathways that the effects of ginsenoside F1 in treating lipid metabolism disorders may be exerted through the regulation of these metabolic pathways.

### 2.7. Network Pharmacological Analysis of Ginsenoside F1 in HepG2 Cells Induced by Oleic Acid and Palmitic Acid

**Figure 10:**
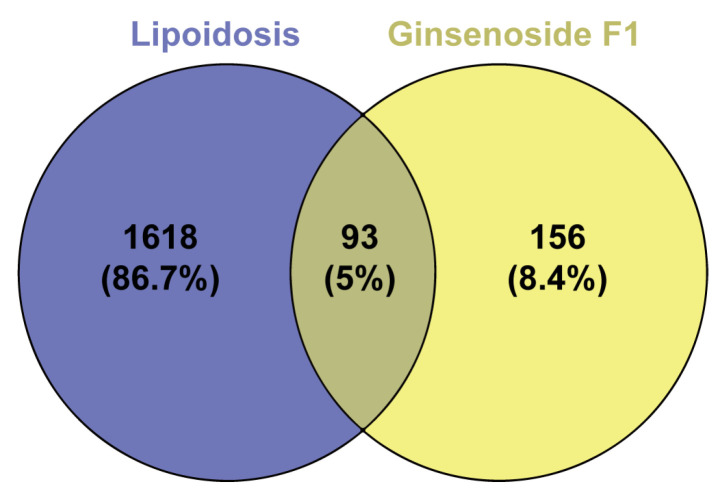
Venn diagram showing the overlap between ginsenoside F1 drug targets and lipid-metabolism-disorder-related gene targets. The blue circle represents genes related to lipoidosis (1618 unique genes, 86.7%). The yellow circle represents potential targets of Ginsenoside F1 (156 unique targets, 8.4%). The overlapping region (greenish color) indicates common targets between the two sets (93 genes, 5%). The percentages refer to the proportion of each category relative to the total number of genes/targets included in the analysis.

**Figure 11:**
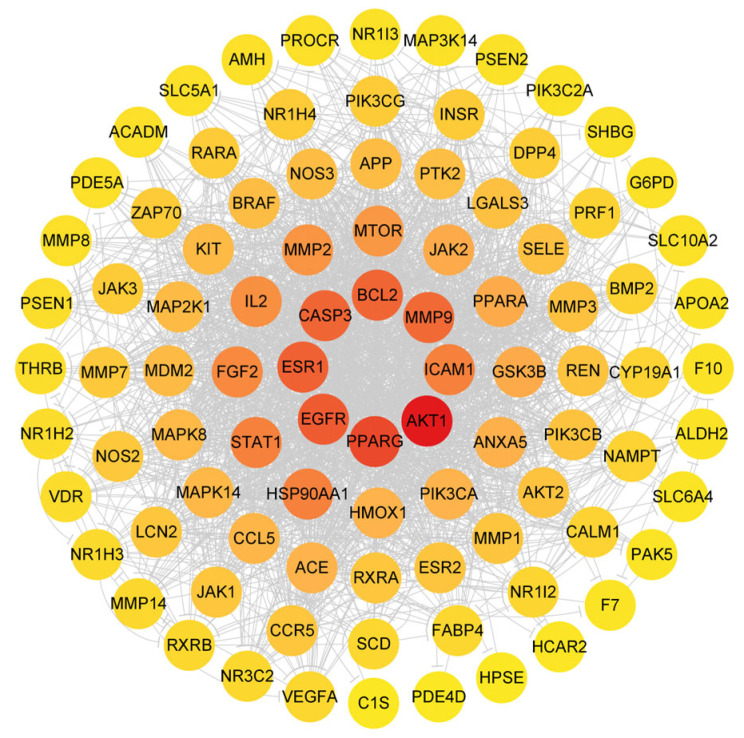
Visualization of PPI network analysis. Nodes represent target proteins, and edges represent their interactions. The color gradient from yellow to red reflects the degree of each node, with red indicating core hub proteins.

**Figure 12:**
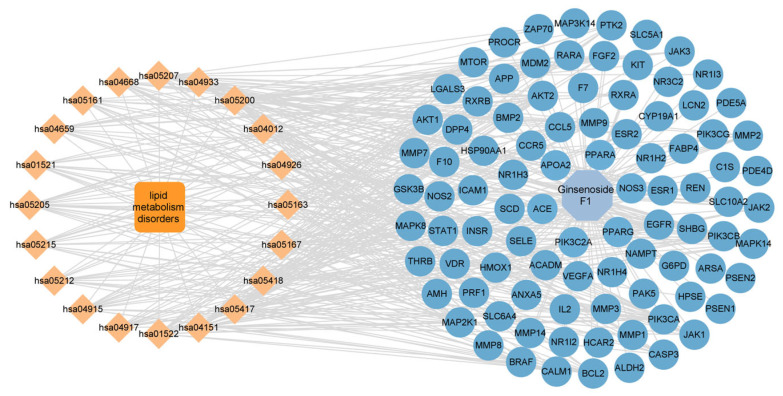
Compound-target-pathway network based on KEGG enrichment analysis. The blue hexagon represents Ginsenoside F1, blue circles represent potential targets, and orange diamonds represent related pathways. The central orange square indicates the core pathway: lipid metabolism disorders.

To investigate the potential effects of ginsenoside F1 on lipid metabolism disorders, a network pharmacology analysis was conducted. Preliminary screening was performed using Venny 2.1 software, through which 93 overlapping targets were identified from 1712 lipid metabolism-disorder-related targets (Figure 10). These overlapping targets were considered as potential targets of ginsenoside F1 in regulating lipid metabolism disorders. A protein–protein interaction (PPI) network was constructed and analyzed using Cytoscape 3.9.1 (The Cytoscape Consortium, San Diego, CA, USA) software, and a compound-target-pathway network was established based on KEGG enrichment analysis (Figure 11 and Figure 12). The top six core targets, including AKT1, PPARG, EGFR, ESR1, CASP3, and BCL2, were screened according to their degree values.

**Figure 13:**
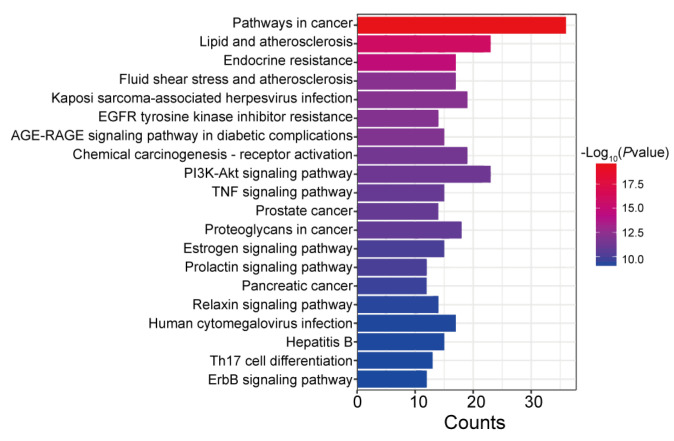
KEGG enrichment analysis of intersection targets between ginsenoside F1 and lipid metabolism disorders.

As illustrated in Figure 13, KEGG enrichment analysis of the overlapping targets revealed that they were significantly enriched in several key pathways, including pathways in cancer, lipid and atherosclerosis, endocrine resistance, fluid shear stress and atherosclerosis, Kaposi sarcoma-associated herpesvirus infection, and so on.

**Figure 14:**
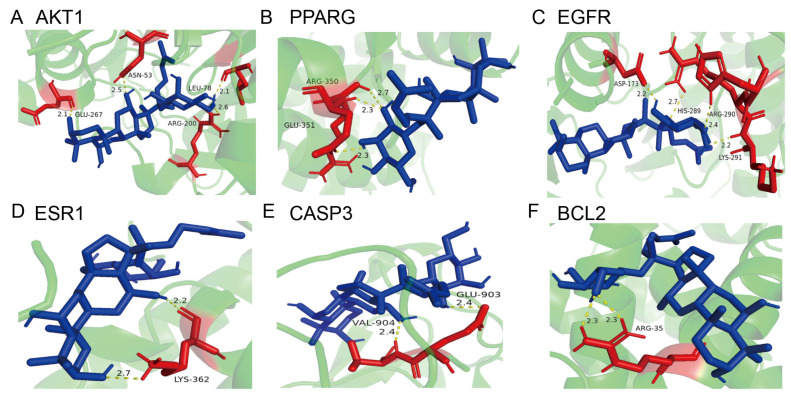
Molecular docking of ginsenoside F1 with six key targets. (**A**) AKT1; (**B**) PPARG; (**C**) EGFR; (**D**) ESR1; (**E**) CASP3; (**F**) BCL2. The blue structure represents Ginsenoside F1, red residues represent key amino acids involved in binding, and green ribbons represent the protein backbone. Yellow dashed lines indicate hydrogen bonds, with numbers showing bond lengths (Å).

Molecular docking analysis was further conducted to explore the binding capacity of ginsenoside F1 with core candidate targets. As shown in Figure 14, the binding affinities of ginsenoside F1 to AKT1, PPARG, EGFR, ESR1, CASP3 and BCL2 were all lower than −5 kcal·mol^−1^, indicating stable and strong binding interactions between the compound and these key proteins. Nevertheless, these in silico docking outcomes are only preliminary computational predictions and still require further in vitro and in vivo experimental validation.

## 3. Discussion

After stimulating HepG2 cells with excessive oleic acid and palmitic acid, HepG2 cells developed severe lipid accumulation, frequently accompanied by significant oxidative stress abnormalities [18,19]. For the simulation of lipid metabolism disorders, an in vitro lipid accumulation model was established using HepG2 in this study. It was demonstrated that significant regulatory effects on the lipid content of the HepG2 cells induced by oleic acid and palmitic acid were observed with 0.8 µM and 3.2 µM ginsenoside F1 (*p* < 0.05), and the relative intracellular TC and TG levels were significantly reduced compared to those in the model group (*p* < 0.05). A certain regulatory effect of ginsenoside F1 on lipid accumulation in HepG2 cells was suggested by the above experimental results.

In the multivariate statistical analysis of lipidomics, it was indicated by the PCA and OPLS-DA results that the experimental method was demonstrated to have high reliability and predictive ability for the detection of differential metabolites. This finding can be used as a reference for the exploration of the mechanism related to the intervention of ginsenoside F1 in lipid metabolism in HepG2 cells induced by oleic and palmitic acid. Using predefined thresholds of VIP > 1, FC ≥ 2 or <0.5, and *p* < 0.05, 110 differential metabolites were screened. Relative to the control group, the model group had 41 substances significantly increased, mainly including phosphatidylcholines, triglycerides, sphingomyelins, and diglycerides. Compared to the model group, a total of 71 substances were significantly downregulated in the ginsenoside F1 group, primarily including fatty acids, ceramides, and phosphatidylcholines. Based on the cluster analysis results of differential metabolites, a tendency of lipid metabolites in HepG2 cells simulating lipid accumulation to revert towards the control group after ginsenoside F1 intervention was observed, which supported the potential regulatory role of ginsenoside F1 on lipid metabolism disorders.

It was revealed by pathway analysis of differential metabolite that the effect of ginsenoside F1 on regulating lipid disorders may be mediated through pathways such as glycerophospholipid metabolism, ether lipid metabolism, glycerolipid metabolism, the phosphatidylinositol signaling system, glycosaminoglycan biosynthesis, sphingolipid metabolism, and linoleic acid metabolism, with the glycerophospholipid metabolism pathway being identified as potentially playing a major role.

Glycerophospholipid metabolism, primarily through the hydrolysis of various phospholipases, ensured normal energy and various metabolic processes in the body, playing a crucial role in regulating intracellular signaling pathways, providing membrane stability, fluidity, and permeability, and in membrane anchoring and substrate transport [20]. As shown in Figure 9 and Table 2, in the glycerophospholipid metabolism pathway, the contents of PC, PI, PE, PS, LPC, PG, and DG significantly changed. Glycerophospholipids comprise phosphatidylglycerol, phosphatidylethanolamine, phosphatidylinositol, phosphatidylcholine, phosphatidylserine, and cardiolipin, among which PC and PE are the two most abundant types in eukaryotic cells and play important roles in cell membranes [21]. Phosphatidylcholine demonstrated therapeutic benefits in improving dyslipidemia and preventing atherosclerosis. It has a positive effect on improving dyslipidemia, especially in reducing cholesterol levels. It has been found by some researchers that abnormal phosphatidylcholine metabolism is closely related to lipid metabolism disorders, and that phosphatidylcholine has a protective effect against lipid disorders [22,23]. Compared to those in the control group, the contents of PC 16:0_16:0, PC 18:0_18:1, PC 16:0_18:0, PC 14:0_16:0, PC 38:4, PC 36:1, PC 15:0_16:0, PC 16:0_17:0, and PC 18:0_22:6 in the model group were significantly upregulated (*p* < 0.05). After ginsenoside F1 intervention, their contents were significantly downregulated (*p* < 0.05) and approached the control levels, further revealing the important role of PC in the improvement of lipid metabolism disorders by ginsenoside F1. After ginsenoside F1 intervention, the PC content was significantly altered and exhibited a trend of reverting towards that in the control group compared to the model group, along with other metabolites in the glycerophospholipid metabolism pathway which were also significantly altered and showed a similar reverting trend. It was thus suggested that these metabolites might be an important material basis for the improvement of lipid metabolism disorders by ginsenoside F1. PI played an important role in cell membrane signal transduction and lipid metabolism regulation, promoting the reverse transport of cholesterol by high-density lipoprotein (HDL) and accelerating the metabolic clearance of cholesterol through the liver, bile, and feces [24]. Compared to that in the control group, a decrease in phosphatidylinositol abundance was demonstrated in the model group cells, suggesting a reduced ability for cholesterol reverse transport and clearance. In contrast, an increase in PI abundance was demonstrated in the ginsenoside F1 group relative to that in the model group. Therefore, it was speculated that ginsenoside F1 might ameliorate lipid disorders by regulating PI levels.

Network pharmacology analysis suggested that a crucial role might be played by the PI3K-Akt signaling pathway in ginsenoside F1-mediated amelioration of disordered lipid metabolism. It was demonstrated in previous studies that the PI3K/AKT signaling pathway is involved in various physiological processes, including cell growth, proliferation, and metabolism, and that the regulation of lipid metabolism is mediated by it. Its downstream key target protein, AKT, a serine/threonine kinase, is closely associated with lipid metabolism [25]. Integrated with metabolomics findings, it was inferred that AKT might be activated by ginsenoside F1 through the phosphorylation of PI via action on the PI3K/Akt signaling pathway, thereby regulating downstream target proteins such as SREBP1 and PPARα to ameliorate abnormal lipid metabolism.

In summary, ginsenoside F1 was postulated to act on targets such as AKT1, PPARG, and EGFR, and to ameliorate lipid disorder in HepG2 cells by the promotion of lipid metabolism and transformation in vivo through influences on multiple pathways including glycerophospholipid metabolism, the PI3K/AKT signaling pathway, ether lipid metabolism, glycerolipid metabolism, the phosphoinositide pathway, glycosaminoglycan biosynthesis, sphingolipid metabolism, and linoleic acid metabolism. However, these conclusions are derived solely from a cell-based model and computational predictions; experimental validation in vivo and at the protein level is required before any definitive claims can be made.

## 4. Materials and Methods

### 4.1. Materials

#### 4.1.1. Cell Line

The human hepatoma cell line HepG2 was obtained from the American Type Culture Collection (ATCC), Manassas, VA, USA, Cat. No. HB-8065 and cultured in high-glucose Dulbecco’s Modified Eagle Medium (DMEM) (Procell Life Science & Technology Co., Ltd., Wuhan, China, Catalog No. WHB824X191), supplemented with 10% (*v*/*v*) fetal bovine serum (FBS, TOCYTO, China, Catalog No. SH2306) and 1% (*v*/*v*) penicillin-streptomycin solution (Procell Life Science & Technology Co., Ltd., Catalog No. PB180120).

#### 4.1.2. Drugs and Reagents

Ginsenoside F1 (≥98% HPLC purity; Chengdu Refines Biology Technology, Chengdu, China; 53963-43-2) and simvastatin (≥98% HPLC purity; APExBIO, Houston, TX, USA; A852231337769) were commercially obtained. Oleic acid, palmitic acid, and BSA (Shanghai Yuanye Bio-Technology, Shanghai, China) were also acquired. The CCK-8 kit and TC/TG assay kits were from Nanjing Jiancheng Bioengineering Institute (Nanjing, China). All chromatographic solvents were supplied by ANPEL Laboratory Technologies (Inc., Shanghai, China) and the certified lipid standard mixture was from Merck KGaA, Darmstadt, Germany.

#### 4.1.3. Instruments

Chromatographic separation and mass spectrometric analysis were conducted on a Nexera UPLC-40DXR system (SHIMADZU, Kyoto, Japan) coupled with an X500R QTOF-MS/MS mass spectrometer (AB Sciex, Framingham, MA, USA). Absorbance measurements were taken with a Multiskan Go full-wavelength microplate reader from Thermo Fisher Scientific (Waltham, MA, USA), and cell counting was performed using an IC-1000 automated cell counter from Countstar (Shanghai, China).

### 4.2. Methods

#### 4.2.1. Determination of the Effect of Ginsenoside F1 on HepG2 Cell Proliferation/Toxicity Using CCK-8 Assay

HepG2 cells were treated with 0.2 µM, 0.8 µM, and 3.2 µM ginsenoside F1 for 24 h to detect its effect on cell viability. Cells were seeded in a 96-well-plate at a density of 5 × 10^5^ cells·mL^−1^, final volume 100 μL, with 6 replicate wells per group. Five groups were set as the control group, model group, and 0.2 µM, 0.8 µM, and 3.2 µM concentration ginsenoside F1 groups.

#### 4.2.2. Determination of the Effect of Ginsenoside F1 on Lipid Level Influenced by HepG2 Cells Induced by Oleic Acid and Palmitic Acid

Referring to the literature method [26], HepG2 cells in the logarithmic growth phase were seeded in 6-well plates at a density of 5 × 10^5^ cells·mL^−1^ with 2 mL medium per well. After 24 h of adherent culture, cells were continuously treated for 24 h with different interventions: control group (0.1% DMSO), model group (FFA mixture), simvastatin group (FFA mixture + 3.2 µ simvastatin), and ginsenoside F1 intervention groups. Ginsenoside F1 was first dissolved in DMSO, and then serially diluted with 2% (*w*/*v*) BSA working solution to prepare final concentrations of 0.2 µM, 0.8 µM and 3.2 µM containing 0.1% DMSO. All ginsenoside F1 working solutions were filtered through a 0.22 µm microporous membrane before use. Cells in the FFA and ginsenoside F1 groups were incubated with FFA mixture supplemented with corresponding concentrations of ginsenoside F1. The FFA mixture consisted of oleic acid and palmitic acid at a 2:1 volume ratio, with a total final concentration of 0.45 mM (0.30 mM OA and 0.15 mM PA) and was prepared in serum-free medium containing 2% (*w*/*v*) BSA. After the above treatment, the culture medium was discarded, and the cells were rinsed twice with pre-cooled PBS. Intracellular lipids have a specific absorption peak at 510 nm, which can be used for quantitative lipid determination. Subsequently, 60% isopropanol was added to lyse the cells, and the absorbance of each well was measured at 510 nm using a microplate reader. The absorbance value of the control group was set as 100%, and the relative intracellular lipid content of each group was calculated for subsequent quantitative analysis of lipid accumulation.

#### 4.2.3. Determination of the Effect of Ginsenoside F1 on TC and TG Content in HepG2 Cells Treated with Oleic Acid and Palmitic Acid

Intracellular TG and TC levels were detected using the single reagent GPO-PAP method according to the respective kit instructions. The OD value of each well was finally measured with a microplate reader at 450 nm. The average OD value of each group was substituted into the formula for calculation as mentioned in the kit instructions. The TG and TC levels of the control group were both set as 100%, and the relative TG and TC contents of each group were calculated.

#### 4.2.4. Untargeted Lipidomic Study

Referring to the literature method [27,28], the MeOH/MTBE/H2O extraction system was used to collect cell samples. To each cell sample, 1 mL of pre-cooled methanol containing internal standards was added. Cells were scraped and transferred to centrifuge tubes. Then, 2.5 mL of methyl tert-butyl ether (MTBE) was added and vortexed, followed by the addition of 750 μL of ultrapure water, and centrifuged at 10,000 rpm at 4 °C for 15 min. The upper hydrophobic phase was collected and dried under a nitrogen stream. The residue was reconstituted in acetonitrile: isopropanol:water (65:30:5, *v*/*v*/*v*, containing 5 mM ammonium acetate) for LC-MS/MS analysis. Equal amounts of lipid extracts from each sample were pooled to prepare quality control (QC) samples. Throughout the instrumental analysis, QC samples were inserted into the analytical sequence at intervals of every 10 real samples to monitor the robustness of the lipidomics analysis process. The liquid chromatography conditions were summarized in Table 3 as follows. For lipidomics analysis, six biological replicates were prepared for each experimental group, and each replicate was analyzed once.

Mass spectrometric detection was performed using an electrospray ionization source in TOF-MS/MS full scan mode. The ion source parameter settings are presented in Table 4.

#### 4.2.5. Network Pharmacology Analysis and Molecular Docking

The targets of ginsenoside F1 were identified using the Traditional Chinese Medicine Systems Pharmacology (TCMSP) database, PubChem, and the SwissTargetPrediction database. Gene targets associated with lipid metabolism disorders were acquired from the Gene Cards and OMIM databases. Overlapping targets were screened using the Venny 2.1.0 tool, and a protein–protein interaction (PPI) network was constructed using Cytoscape software (version 3.9.1). Core targets were subsequently screened based on topological parameters, including betweenness centrality, closeness centrality, and degree centrality. KEGG pathway enrichment analyses were performed using the Database for Annotation, Visualization and Integrated Discovery (DAVID) (version 6.8). Furthermore, the results of KEGG enrichment analyses were visualized using the bioinformatics platform. Molecular docking was carried out using AutoDock Vina 1.5.6 (The Scripps Research Institute, La Jolla, CA, USA). The 3D structures of target proteins (AKT1, PPARG, EGFR, ESR1, CASP3, BCL2) were obtained from the Protein Data Bank (PDB), and the structure of ginsenoside F1 was retrieved from PubChem. Proteins were prepared by removing water molecules and adding polar hydrogens. The grid box was centered on the reported active site or a co-crystallized ligand, with dimensions set to cover the entire binding cavity. Docking utilized the default scoring function of AutoDock Vina, generating nine binding poses per ligand; the lowest-energy pose was selected. A binding energy below −5 kcal·mol^−1^ was interpreted as strong binding, based on standard criteria. Results were visualized with PyMOL 4.6 (Schrödinger, Inc., New York, NY, USA).

#### 4.2.6. Data Processing

Raw data files were processed using MSDIAL 4.9.1 software for baseline filtering, peak identification, peak alignment, and other preprocessing steps to obtain information such as mass-to-charge ratio and retention time [29]. MSDIAL software was used to integrate and identify the peaks detected in the samples, finally obtaining quantitative results.

The processed data were imported into MetaboAnalyst 6.0 (https://www.metaboanalyst.ca/) accessed on 17 August, 18 August and 21 August 2025. Principal component analysis (PCA) and orthogonal partial least squares-discriminant analysis (OPLS-DA) were used to visualize metabolic differences between experimental groups. Metabolites with a Variable Importance in the Projection (VIP) > 1, Fold Change (FC) ≥ 2 or <0.5, and *p* < 0.05 were screened as potential biomarkers for inter-group differences [30]. Finally, metabolic pathway enrichment analysis of the differential lipid metabolites was performed based on the Kyoto Encyclopedia of Genes and Genomes (KEGG) database.

#### 4.2.7. Statistical Analysis

The experimental data were statistically analyzed using GraphPad Prism software (Ver. 9.0) by one-way analysis of variance (ANOVA). Following one-way ANOVA, multiple comparisons were conducted. Provided that the assumption of homogeneity of variance was met, the *LSD* test was selected. Results were typically reported as *p* < 0.05 (significant), *p* < 0.01 (highly significant), and *p* < 0.001 (extremely significant).

## 5. Conclusions

Ginsenoside F1 was suggested to ameliorate FFA-induced lipid accumulation in HepG2 cells. Lipidomics and network pharmacology analysis indicated that this effect may involve the glycerophospholipid metabolism pathway, the PI3K/AKT signaling pathway, and core targets such as AKT1, along with changes in differential lipids (e.g., PC, PI, DG, LPC). This study provides a theoretical basis for further investigation into the lipid-modulating activity of ginsenoside F1.

However, several key limitations of the present study should be clearly acknowledged. All experiments were performed solely using an FFA-stimulated HepG2 cell model, which only partially mimics abnormal lipid metabolism in hepatocytes and cannot recapitulate the complex pathological features of in vivo systemic hyperlipidemia or liver metabolic disorders. Accordingly, the relevant results and mechanistic speculations are in vitro and preliminary, and the potential application of ginsenoside F1 in improving lipid metabolic disorders should not be overextended. Further rigorous validation, including in vivo animal models, molecular target verification via Western blotting or siRNA interference, and pathway rescue experiments, is therefore required to clarify the exact regulatory mechanism and translational value of ginsenoside F1 in future research.

## Figures and Tables

**Table 1:** OPLS-DA model parameters of cell samples under positive and negative ion modes.

Model Number	Pattern	Sample Group	*R* ^2^ *X*	*R* ^2^ *Y*	*Q* ^2^
A	Positive ion	Control group vs. model group	0.907	1	0.999
B	Positive ion	0.2 μM F1 group vs. model group	0.968	1	1
C	Positive ion	0.8 μM F1 group vs. model group	0.967	1	1
D	Positive ion	3.2 μM F1 group vs. model group	0.971	1	1
E	Negative ion	Control group vs. model group	0.236	0.671	0.518
F	Negative ion	0.2 μM F1 group vs. model group	0.297	0.85	0.727
G	Negative ion	0.8 μM F1 group vs. model group	0.27	0.855	0.694
H	Negative ion	3.2 μM F1 group vs. model group	0.454	0.886	0.846

**Table 2:** Analysis of glycerophospholipid metabolism pathway-related metabolites.

KEGG ID	Class	Metabolite Name	Model vs. Control	F1 vs. Model
C00157	Phosphatidylcholine	PC 16:0_16:0	↑	↓
		PC 18:0_18:1	↑	↓
		PC 16:0_18:0	↑	↓
	Phosphatidylcholine	PC 36:1	↑	↓
		PC 14:0_16:0	↑	↓
		PC 38:4	↑	↓
		PC 15:0_16:0	↑	↓
		PC 16:0_17:0	↑	↓
		PC 18:0_22:6	↑	↓
C00641	Diglyceride	DG 34:0	↑	↓
		DG 32:0	↑	↓
		DG 16:0_18:0	↑	↓
		DG 37:7	↑	↓
		DG 36:0	↑	↓
		DG 18:0_18:0	↑	↓

These metabolites were involved in glycerophospholipid metabolism, offering deeper insights into the protective mechanism against lipid metabolism disorders by ginsenoside F1. ↑ indicates significant upregulation (*p* < 0.05); ↓ indicates significant downregulation (*p* < 0.05).

**Table 3:** Liquid chromatography parameters.

	Mobile Phase Conditions	
Instrument	Nexera UPLC-40DXR system (SHIMADZU, Japan)	
Column	ACQUITY UPLC BEH C_18_ column (2.1 mm × 100 mm, 1.7 μm, Waters)	
Column temperature	37 °C	
Mobile phase A	Acetonitrile-ultrapure water mixture (6:4, *v*/*v*)	
Mobile phase B	Isopropanol-acetonitrile mixture (9:1, *v*/*v*)	
Gradient program	0–2 min	Mobile phase B, 10–40%
	2–5 min	Mobile phase B, 40–46%
	5–15 min	Mobile phase B, 46–48%
	15–16.5 min	Mobile phase B, 48–100%
	36.8–38.5 min	Mobile phase B, 10%
Flow rate	0.26 mL·min^−1^	
Injection volume	2 μL	

**Table 4:** Mass spectrometer parameters.

	Positive Ion Mode	Negative Ion Mode
Spray voltage (V)	5500	−4500
Nebulizer gas pressure (psi)	50	50
Auxiliary/dry gas pressure (psi)	50	50
Curtain gas pressure (psi)	30	30
Ion source dry gas temperature (°C)	500	500
Collision gas pressure (psi)	7	7
TOF start mass (Da)	50	50
TOF end mass (Da)	1200	1200
Declustering potential (V)	60	60
Accumulation time (s)	0.15	0.15

## Data Availability

The metabolomics data have been deposited to MetaboLights [31] repository with the study identifier MTBLS13976.
